# Single-cell reanalysis identifies macrophage-associated transcriptional and intercellular communication features in diabetic foot ulcers

**DOI:** 10.3389/fcimb.2026.1875481

**Published:** 2026-07-01

**Authors:** Jia Yao, Chang-qing Zhu, Xue-jian Deng, Jiong-biao Yu, Tuo-pu Wu, Yan Sun, Wan-mei Li

**Affiliations:** 1 Department of Endocrinology, Guangzhou Twelfth People’s Hospital, Guangzhou, China; 2 The Affiliated Dongguan Songshan Lake Central Hospital, Guangdong Medical University, Dongguan, China; 3 The First Affiliated Hospital of Guangdong Pharmaceutical University, Guangzhou, China

**Keywords:** diabetic foot ulcer, diabetic non-ulcer foot skin, macrophage, pseudobulk differential expression, single-cell RNA sequencing

## Abstract

**Background:**

Diabetic foot ulcers (DFUs) are devastating diabetic complications characterized by an extremely high non-healing rate and elevated risk of life-threatening outcomes. To characterize the cellular landscape and macrophage-associated alterations between DFU tissue and diabetic non-ulcer foot (DNFU) skin, we performed a comprehensive reanalysis of publicly available single-cell RNA sequencing (scRNA-seq) datasets.

**Methods:**

A fully reproducible R-based analytical pipeline was used to process public scRNA-seq count matrices (GSE165816) from 5 DFU and 8 DNFU skin samples. A total of 27,844 cells (16,579 from DFU and 11,265 from DNFU skin) passed stringent quality control filters. Data normalization, integration, dimensionality reduction, unsupervised clustering, and marker-based cell type annotation were performed using the Seurat and Harmony packages. Sample-level comparisons included cell fraction analysis and UCell pathway scoring. Macrophages were further investigated through subclustering analysis, edgeR-based pseudobulk differential expression analysis, volcano plot visualization, GO over-representation analysis, enrichment analysis, and CellChat ligand-receptor interaction inference.

**Results:**

The integrated single-cell atlas resolved 10 major cell compartments including epithelial, stromal, endothelial, lymphatic endothelial, smooth muscle/pericyte, lymphoid, myeloid, mast cell, plasma cell, and melanocyte/Schwann cells. Sample-level cell fraction analysis revealed a nominal reduction in lymphatic endothelial cells in DFU; however, no cell type proportion differences remained statistically significant after false discovery rate (FDR) correction. Similarly, no significant differences were observed in macrophage UCell pathway scores for glycolysis, lactate metabolism, combined glycolysis-lactate metabolism, TNF-NF-κB signaling, or insulin/IGF signaling between DFU and DNFU skin following FDR correction. Macrophage subclustering identified DFU-enriched subpopulations, and macrophage pseudobulk analysis prioritized multiple DFU-associated genes. Notably, sex-linked genes including XIST, RPS4Y1, and DDX3Y reached statistical significance after FDR correction, indicating that a portion of the observed transcriptional signal may be attributed to demographic imbalance between groups. Exploratory enrichment analysis of nominal and ranked macrophage differentially expressed genes highlighted DFU-directional enrichment of translation, oxidative phosphorylation, electron transport, and antigen presentation terms, while DNFU-directional genes were enriched for transcriptional regulation and rhythmic process terms. CellChat analysis prioritized macrophage-associated CD99-CD99 and MIF-CD74/CXCR4 ligand-receptor pairs as key DFU-associated intercellular communication hypotheses.

**Conclusion:**

This single-cell reanalysis comparing DFU and DNFU skin identifies putative ligand-receptor interaction hypotheses, transcriptomic alterations, and macrophage subpopulation shifts that require definitive validation in larger, clinically well-characterized, and spatially resolved patient cohorts.

## 1 Introduction

Diabetic foot ulcers (DFU) are devastating diabetic complications associated with increased risks of life-threatening infection, non-traumatic amputation, ulcer recurrence, and mortality, and impose a growing socioeconomic burden on global healthcare systems (Darwin and Tomic-Canic, 2018; Gomes et al., 2022). A significant portion of DFUs develop into chronic, non-healing wounds resistant to standard-of-care interventions, despite the fact that current international clinical guidelines place a high priority on evidence-based strategies such as preventive foot care, thorough vascular assessment, targeted antimicrobial therapy, pressure offloading, sharp debridement, and multidisciplinary team management (Al-Jalodi et al., 2022). This significant clinical variability highlights the intricate, multifactorial pathobiology of DFUs, which is fueled by aberrant and dysregulated crosstalk between immune, vascular, stromal, neural, and epithelial compartments within the wound microenvironment rather than a single dysregulated pathway.

An essential part of physiological tissue repair, acute, self-resolving inflammation has been retained throughout evolution. On the other hand, chronic wound states are caused by persistent, non-resolving inflammatory signaling cascades that interfere with restorative processes (Eming et al., 2017). All stages of wound healing are orchestrated by macrophages, which mediate effective debris removal, temporal regulation of inflammatory amplification and resolution, angiogenesis, extracellular matrix remodeling, and bidirectional communication with fibroblasts, endothelial cells, and keratinocytes (Eming et al., 2017; Krzyszczyk et al., 2018). Crucially, single-cell RNA sequencing (scRNA-seq) has emerged as a revolutionary technology to analyze disease-associated macrophage heterogeneity and tissue-context-dependent intercellular communication states at single-cell resolution. The oversimplified M1/M2 polarization paradigm cannot adequately capture macrophage biology in human chronic wounds.

Although scRNA-seq has transformed our comprehension of the cellular complexity of human DFU tissue by identifying immune cell subsets and wound-region-specific stromal transcriptional programs linked to different healing outcomes (Theocharidis et al., 2022). Studies restricted exclusively to ulcerated tissue cannot disentangle DFU-specific pathobiology from the baseline molecular alterations intrinsic to diabetic skin, sampling site variability, and inter-individual patient heterogeneity. Therefore, attributing DFU specificity to observed cellular and molecular phenotypes necessitates rigorous, disease-relevant comparisons with diabetic non-ulcer foot (DNFU) skin.

Using currently available public single-cell transcriptomic resources, this study attempts to systematically analyze macrophage-centered cellular heterogeneity and molecular regulatory features in the diabetic foot ulcer microenvironment through a comparison between DFU tissue and DNFU skin. Our goal is to find intercellular communication axes and high-priority candidate genes linked to the pathophysiology of DFU. The macrophage subpopulation shifts, differential transcriptional signatures, and important cell-cell interaction patterns found here offer a solid foundational framework for future large-scale clinical cohort validation, spatial transcriptomic *in situ* localization, and *in vitro*/*in vivo* functional mechanistic investigations, even though the small sample size and lack of clinical metadata of the publicly available datasets prevent definitive causal mechanistic conclusions. In order to clarify the immunopathological foundation of chronic non-healing DFUs and inform future targeted therapeutic strategies, this work establishes preliminary evidence.

## 2 Materials and methods

### 2.1 Data source and group definition

The public DFU single-cell resource described by Theocharidis et al. (2022) (GSE165816) provided the raw scRNA-seq count matrices. Samples classified as DFU tissue and DNFU skin were used in this analysis. Five DFU samples and eight DNFU skin samples were used in the final comparison. To facilitate sample-level statistics, the disease group and sample identification were maintained throughout the analysis.

### 2.2 Quality control, integration, clustering, and annotation

Count matrices were parsed into sample-specific Seurat objects. Cells were retained using nFeature_RNA >= 200, nCount_RNA >= 500, mitochondrial transcript percentage <= 20%, and sample-specific upper quantile filters for nFeature_RNA and nCount_RNA. After QC, 27,844 cells were retained, including 16,579 DFU cells and 11,265 DNFU skin cells. Normalization, variable-feature selection, dimensionality reduction, clustering, and visualization were performed in Seurat (Stuart et al., 2019). Harmony integration was applied using sample_id as the batch variable (Korsunsky et al., 2019). Cell types were annotated using canonical marker genes and exported cluster marker tables.

### 2.3 Cell-composition analysis

Cell fractions were calculated within each sample by dividing the number of cells assigned to each annotated cell type by the total number of retained cells from that sample. DFU and DNFU groups were compared using sample-level Wilcoxon rank-sum tests. Benjamini-Hochberg false discovery rate (FDR) correction was applied across tested cell types.

### 2.4 UCell pathway scoring

UCell was used to calculate rank-based single-cell gene-set scores, a strategy designed to reduce dependence on dataset composition and size (Andreatta and Carmona, 2021). We scored HALLMARK GLYCOLYSIS, HALLMARK TNFA SIGNALING VIA NFKB, a custom lactate metabolism set, a combined glycolysis-lactate metabolism signature (GG Lac), and a custom insulin/IGF signaling set. Sample-level median scores were compared between groups using Wilcoxon tests followed by FDR correction.

### 2.5 Macrophage subclustering

Cells annotated as macrophages were subsetted for focused analysis. Macrophage cells were renormalized, reclustered using 1,500 variable genes and the first 15 principal components and visualized by Uniform Manifold Approximation and Projection (UMAP). Subcluster markers, sample-level subcluster fractions, and DFU/DNFU enrichment ratios were exported.

### 2.6 Macrophage pseudobulk differential expression, volcano visualization, and enrichment analysis

Macrophage raw counts were aggregated by sample for pseudobulk differential expression. Samples with at least 15 macrophage cells were retained. edgeR quasi-likelihood testing was used to compare DFU with DNFU skin (Robinson et al., 2010). This sample-level strategy was chosen because pseudobulk approaches can reduce false discoveries caused by treating individual cells as independent biological replicates (Squair et al., 2021). Genes were ranked by P value for visualization, and the top 20 genes were displayed as sample-level pseudobulk logCPM row z-scores. FDR-adjusted values are reported.

Macrophage pseudobulk differential expression (DE) results were visualized using a volcano plot. Nominal DFU-up and DNFU-up genes were analyzed by GO over-representation analysis using clusterProfiler and org.Hs.eg.db, with all mapped tested macrophage genes as the background (Yu et al., 2012). Ranked Gene Ontology (GO), Biological Process (BP), and gene set enrichment analysis (GSEA) was performed on the full list of mapped genes ranked by signed -log_10_(P value), where positive values indicated genes upregulated in DFU and negative values indicated genes upregulated in DNFU. An additional targeted GSEA was conducted using the identical custom gene sets employed for UCell pathway scoring.

### 2.7 Cell-cell communication inference

CellChat was used to infer ligand-receptor communication probabilities from normalized expression and annotated cell labels (Jin et al., 2021). Complete interaction tables and top differential ligand-receptor pairs were exported.

### 2.8 Statistical analysis

All statistical analyses were performed in R (version 4.3). All analyses were performed in R with fixed random seed. Unless otherwise stated, pairwise group comparisons were conducted using two-sided Wilcoxon rank-sum tests. For cell−composition analysis and UCell pathway scoring, the sample−level summary measure (fraction for composition; median score for UCell) was compared between DFU and DNFU groups. Multiple−comparison adjustment was performed using the Benjamini−Hochberg FDR procedure across all tested cell types (for composition) or across all pathway scores within a given cell population. An FDR−adjusted P value < 0.05 was considered statistically significant. For macrophage pseudobulk differential expression analysis, edgeR quasi−likelihood F−tests were used and genes were considered significantly differentially expressed when FDR < 0.05 and |log_2_ fold change| >= 1. Exploratory enrichment analyses (GO over−representation and GSEA) were conducted on nominal differential expression results (unadjusted P < 0.05, |log_2_ fold change| >= 0.5).

## 3 Results

### 3.1 Integrated skin atlas of DFU versus DNFU

**Figure 1:**
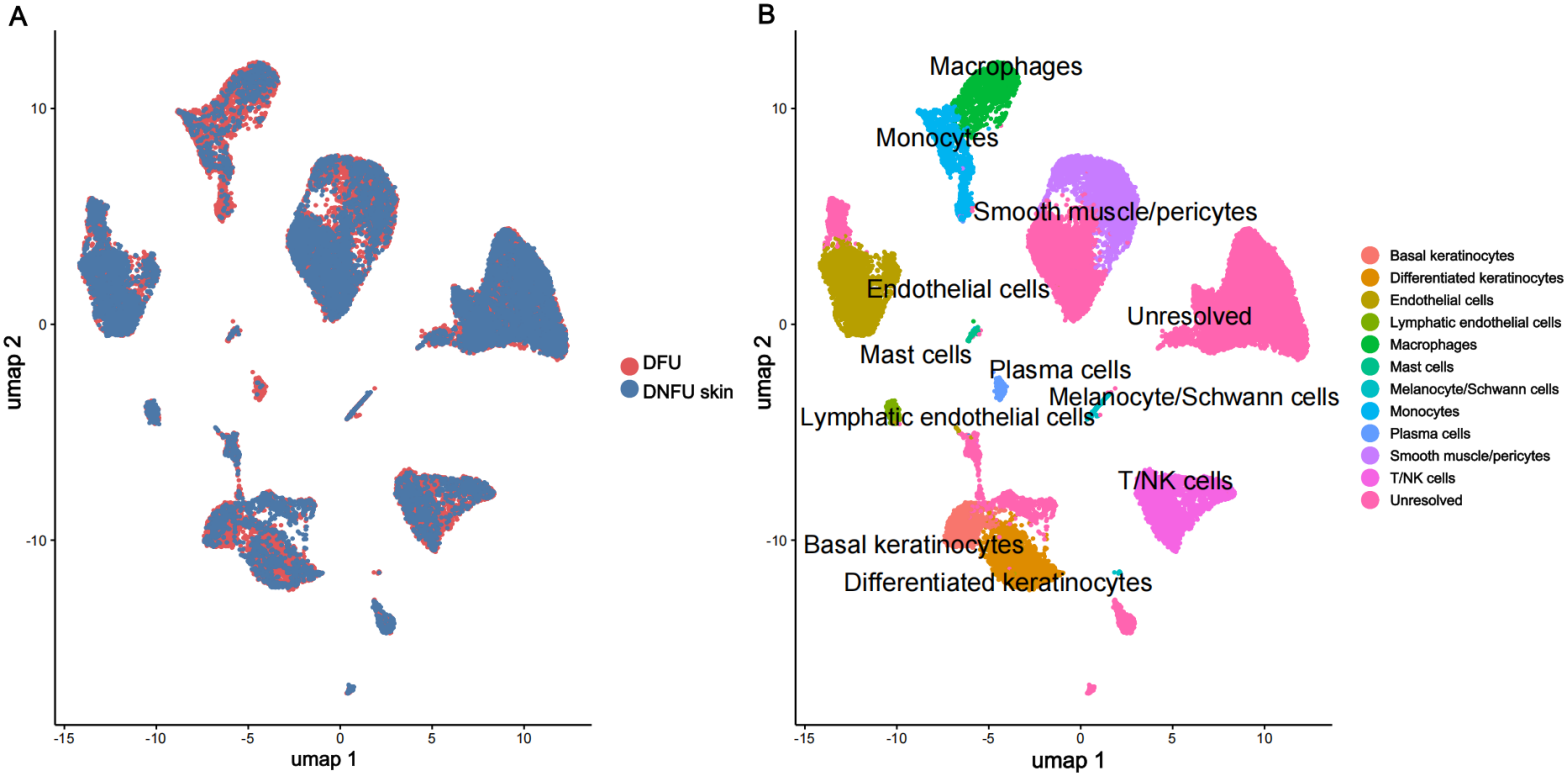
Integrated single-cell transcriptomic atlas of DFU and DNFU skin after Harmony batch correction. The integrated atlas comprised 27,844 quality-controlled cells from 13 samples, including 5 DFU samples (16,579 cells) and 8 DNFU skin samples (11,265 cells). Harmony integration was performed using sample_id as the batch variable after Seurat normalization, variable-feature selection, scaling, and PCA. **(A)** UMAP plot colored by sample group (DFU versus DNFU skin). **(B)** UMAP plot colored by annotated cell types.

**Figure 2:**
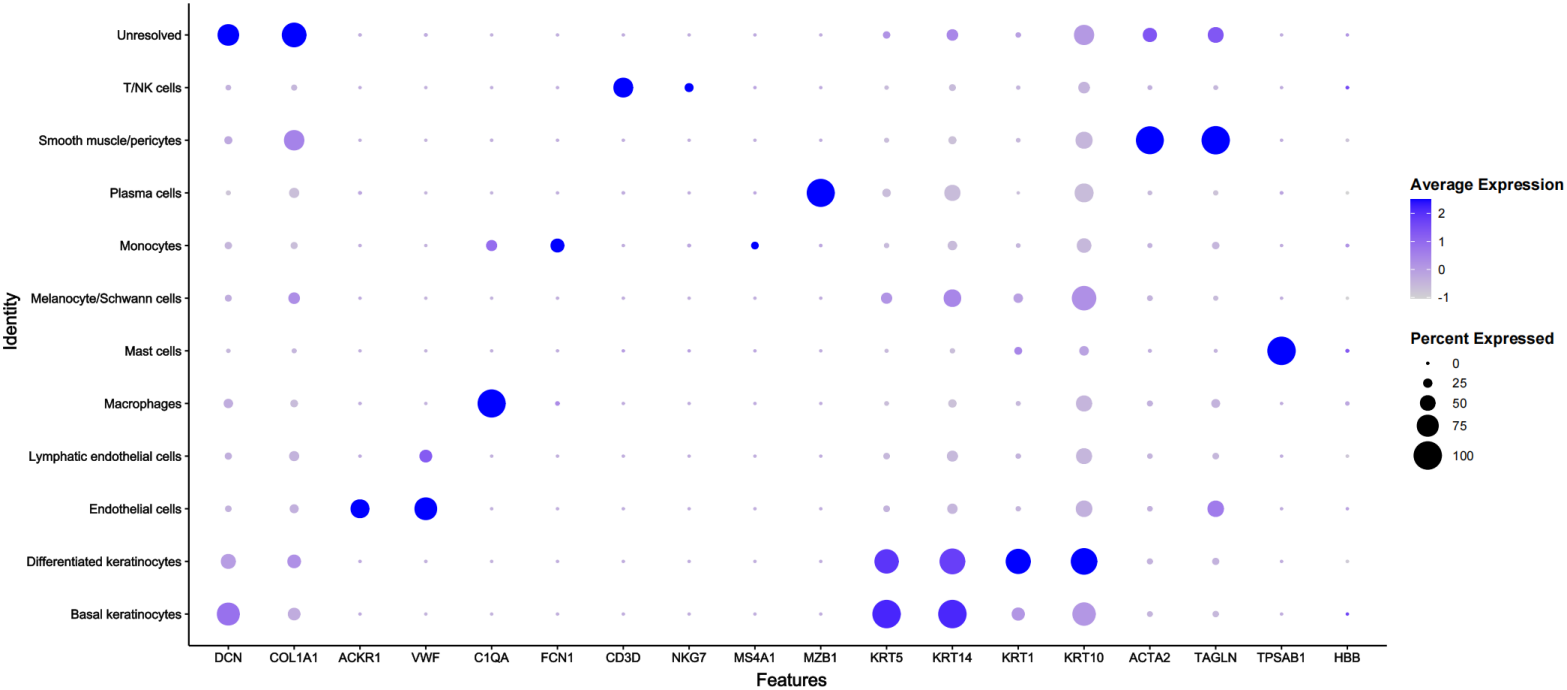
Canonical marker validation of annotated cell types. Dot plot showing canonical marker expression across the annotated cell populations in the integrated atlas of 27,844 cells from 5 DFU and 8 DNFU skin samples. Dot size indicates the percentage of cells expressing each marker within the indicated cell population, and color intensity indicates the scaled average expression level. Cell-type annotation was assigned after graph-based clustering of the Harmony-integrated dataset.

Following quality control, 27,844 cells total—16,579 from DFU samples and 11,265 from DNFU skin samples—were included in the integrated dataset (**Supplementary Figure 1**). While maintaining discrete epithelial, stromal, vascular, immunological, and neural-associated compartments, UMAP imaging revealed widespread integration between groups (**Figure 1**). Basal keratinocytes, developed keratinocytes, endothelial cells, lymphatic endothelial cells, smooth muscle/pericytes, monocytes, macrophages, T/NK cells, mast cells, plasma cells, melanocyte/Schwann cells, and unresolved cells were all resolved by marker-based annotation. The annotation system was supported by canonical marker expression (**Figure 2**).

### 3.2 Cell-composition differences

**Figure 3:**
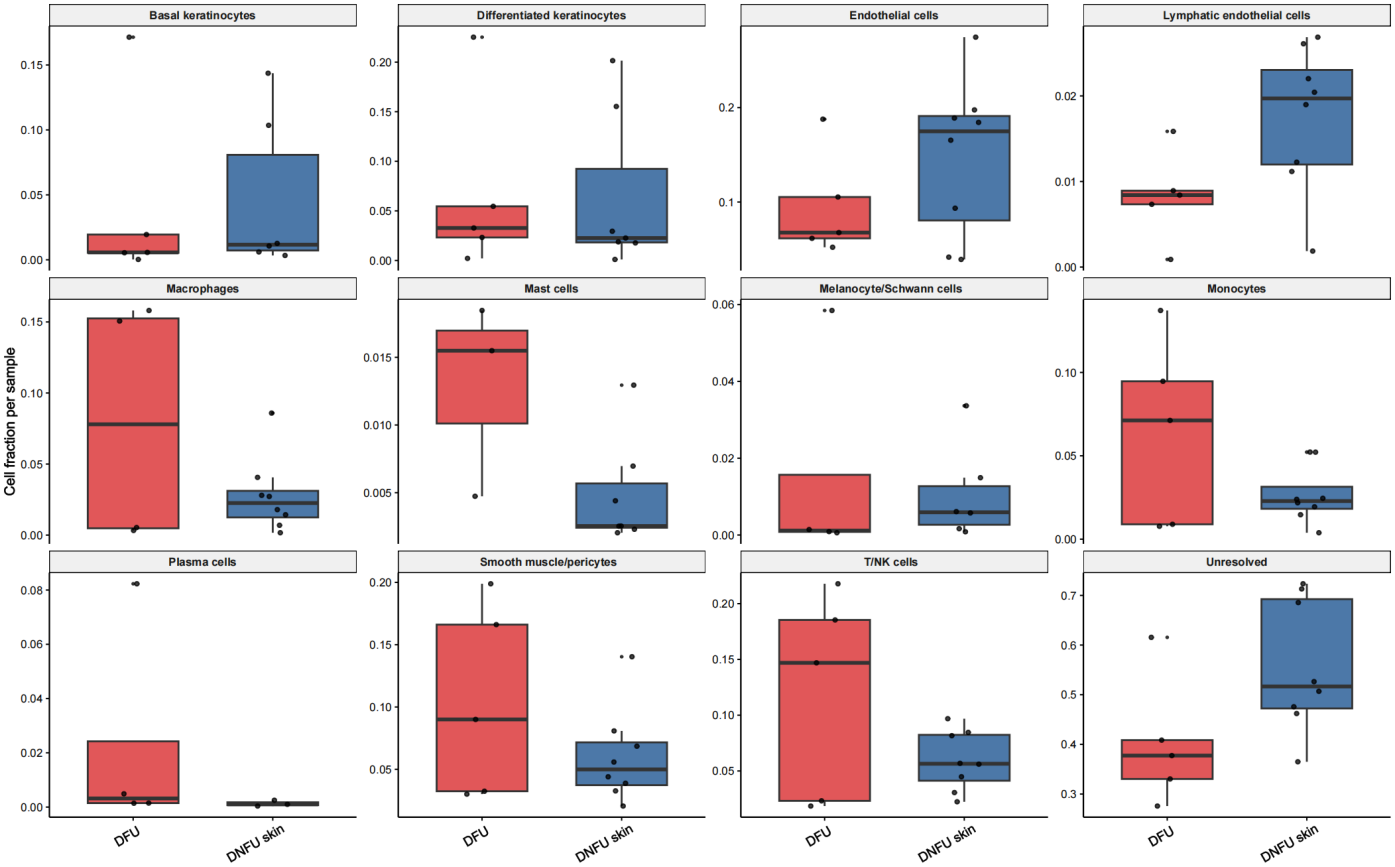
Sample-level cell-composition comparison between DFU and DNFU skin. Box plots show per-sample cell fractions for each annotated cell population across 13 samples (5 DFU and 8 DNFU skin samples). Each point represents one sample. Group comparisons were performed between DFU and DNFU skin at the sample level using two-sided Wilcoxon rank-sum tests, followed by Benjamini-Hochberg false-discovery-rate correction.

Nominal differences between DFU and DNFU skin were shown by sample-level cell-composition analysis (**Figure 3**). DFU skin had fewer lymphatic endothelial cells than DNFU skin (median 0.0084 vs. 0.0197; Wilcoxon P = 0.048, FDR = 0.273). Endothelial cells and melanocyte/Schwann cells tended to be lower in DFU, while mast cells, monocytes, smooth muscle/pericytes, and T/NK cells tended to be higher. These findings should be regarded as tissue-context trends rather than definitive cell-composition changes since none of the cell-fraction comparisons remained significant after FDR correction.

### 3.3 Pathway scores

**Figure 4:**
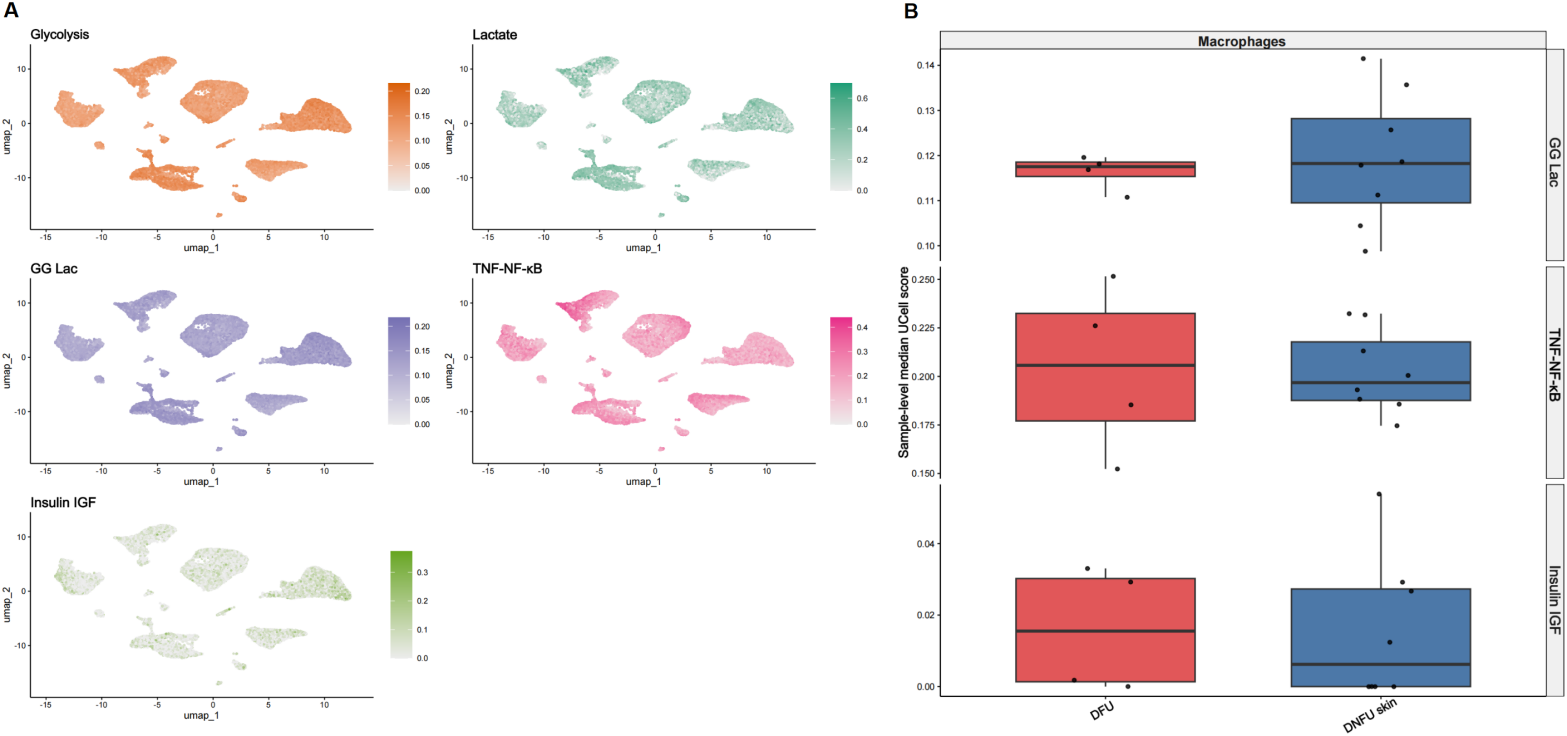
UCell pathway scores in the DFU-vs-DNFU skin atlas and macrophages. UCell scoring was performed on the integrated atlas of 27,844 cells from 5 DFU and 8 DNFU skin samples using predefined glycolysis, lactate metabolism, GG Lac, TNF-NF-κB, and insulin/IGF gene signatures. **(A)** UMAP feature plots showing per-cell UCell scores across the full integrated atlas. **(B)** Sample-level median macrophage UCell scores comparing DFU and DNFU skin. Only samples containing macrophages were included in panel B (4 DFU and 8 DNFU skin samples). Each point represents one sample. Group comparisons were performed using two-sided Wilcoxon rank-sum tests with Benjamini-Hochberg false-discovery-rate correction.

Glycolysis, lactate metabolism, GG Lac, TNF-NF-κB, and insulin/IGF signature activity varied across the integrated atlas, according to UCell maps (**Figure 4A**). However, following FDR correction, there was no significant difference in macrophage sample-level median scores between DFU and DNFU skin (**Figure 4B**). While GG Lac, lactate metabolism, TNF-NF-κB, and glycolysis scores were comparable between groups, macrophage insulin/IGF scores were numerically higher in DFU than in DNFU skin, but this difference was not statistically significant (median 0.0155 vs. 0.0062; P = 0.538, FDR = 1). Consequently, the pathway-score analysis yields a negative or non-significant result that limits the strength of downstream interpretation rather than demonstrating macrophage pathway activation in DFU.

### 3.4 Subclustering

**Figure 5:**
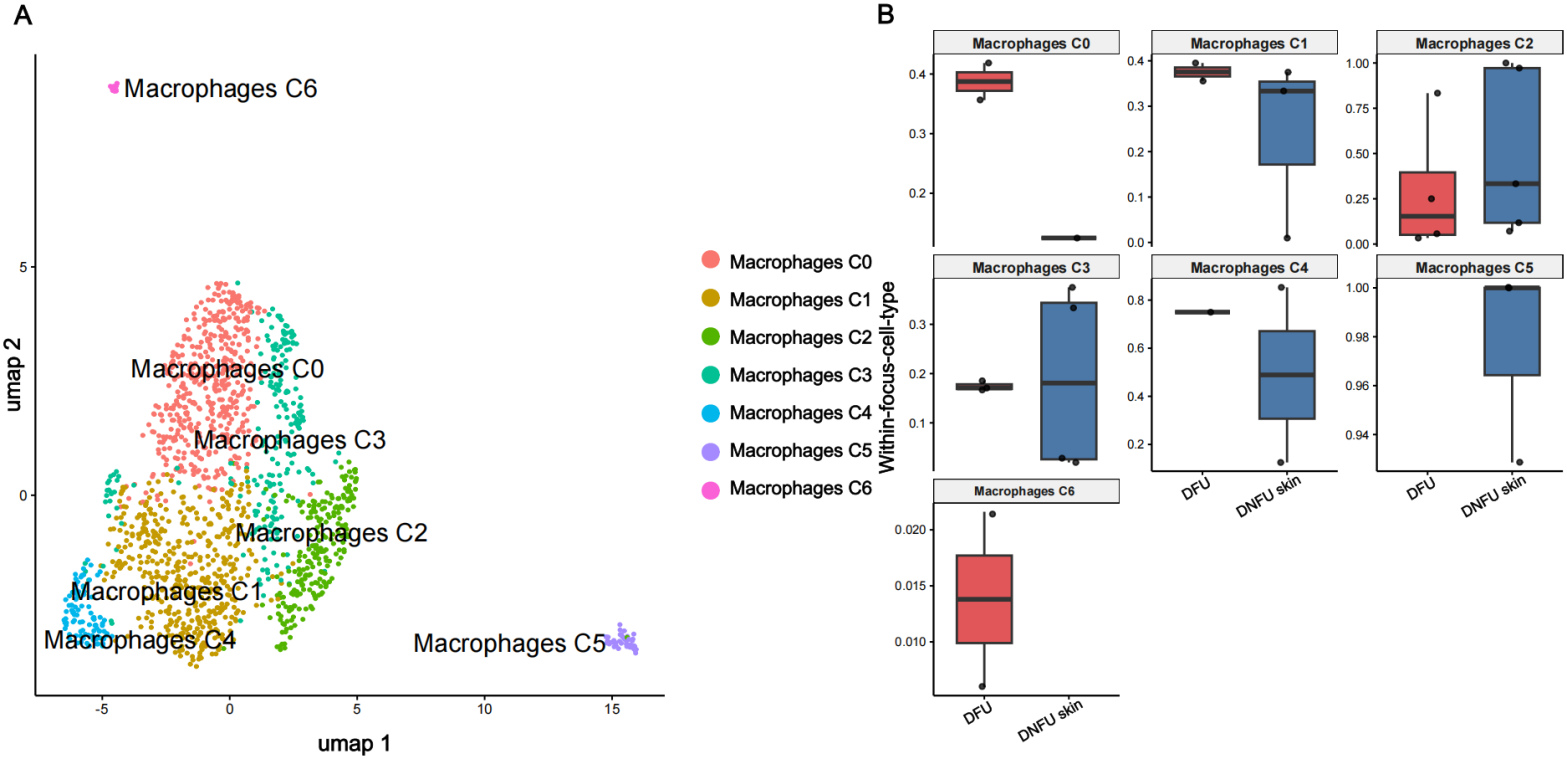
Macrophage subclustering. Macrophage subclustering was performed on 1,508 macrophages identified in the integrated atlas (1,197 DFU and 311 DNFU skin macrophages). Seven macrophage subclusters were identified by graph-based clustering after macrophage-specific reprocessing. **(A)** UMAP showing macrophage subcluster structure. **(B)** Box plots showing sample-level within-macrophage subcluster fractions in DFU versus DNFU skin. Samples contributing macrophages to this analysis included 4 DFU and 8 DNFU skin samples. Each point represents one sample. These plots show descriptive between-group distributions of macrophage subcluster composition.

Seven macrophage subclusters were found using macrophage-focused subclustering (**Figure 5**). Macrophages C0 and C4 were comparatively enriched in DFU, according to sample-level enrichment analysis, with median enrichment ratios of roughly 3.10 and 1.53, respectively. In DNFU skin, macrophages C2 and C5 were comparatively more prevalent. These results suggest that the predominant macrophage-associated signal in this analysis is a shift in macrophage subpopulation composition, rather than differential pathway activation.

### 3.5 Pseudobulk analysis

**Figure 6:**
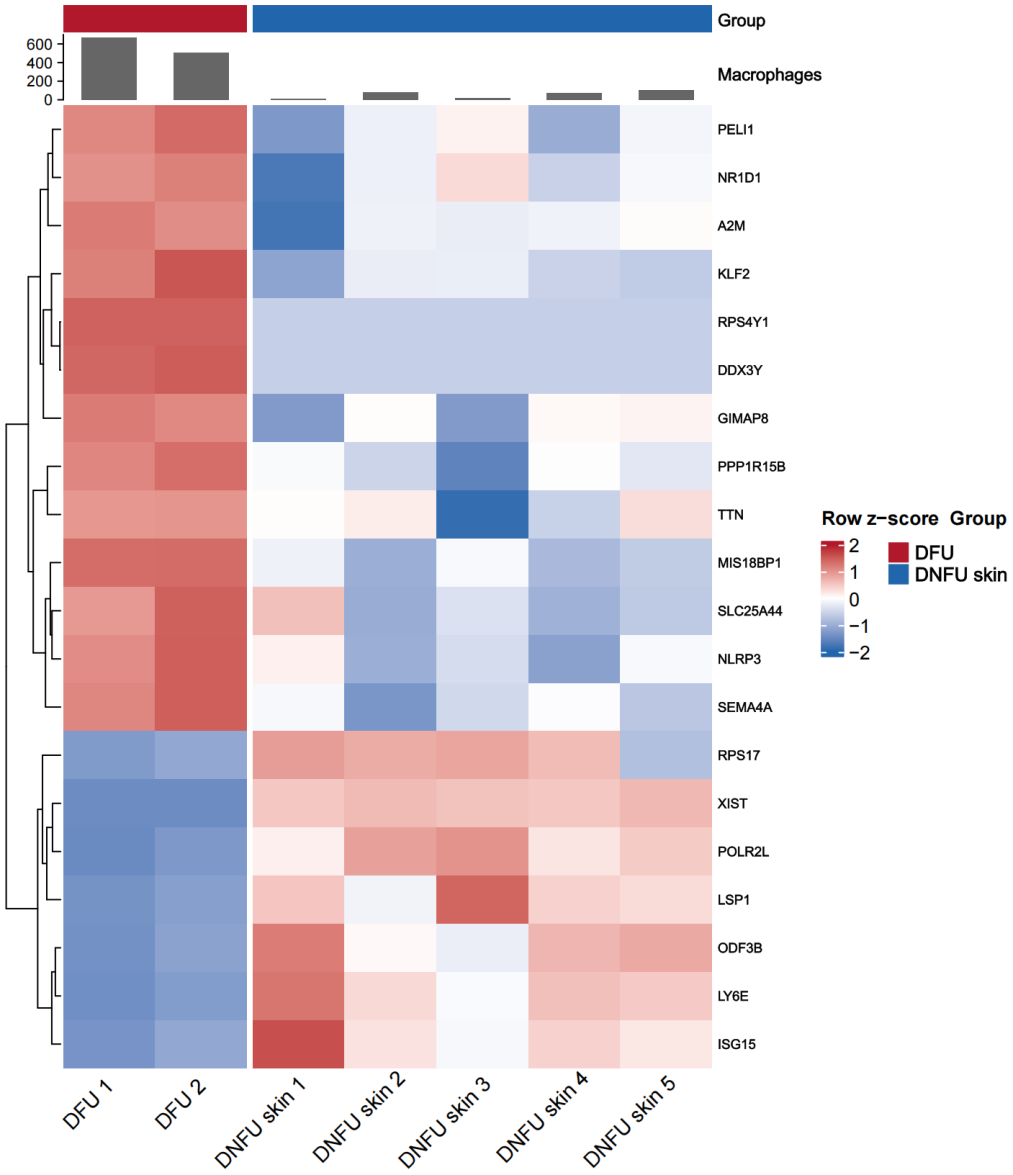
Macrophage pseudobulk differential expression heatmap. Heatmap of representative macrophage pseudobulk differential-expression genes for the DFU-versus-DNFU skin comparison. Pseudobulk differential expression was performed with edgeR using macrophage-containing samples that met the prespecified minimum threshold of 15 macrophages per sample, yielding 7 samples in total (2 DFU and 5 DNFU skin samples). Genes shown were selected from the top-ranked macrophage differential-expression results, and expression values are displayed as scaled expression across pseudobulked macrophage profiles.

3,850 genes were evaluated for macrophage pseudobulk differential expression. For the top 20 genes listed by P value, the heatmap shows sample-level pseudobulk expression patterns (**Figure 6**). FDR significance was attained by XIST (logFC = 12.32, FDR = 1.75e-5), RPS4Y1 (logFC = -11.77, FDR = 0.0017), and DDX3Y (logFC = -10.63, FDR = 0.0067). SLC25A44, LY6E, LSP1, SEMA4A, POLR2L, PPP1R15B, GIMAP8, PELI1, A2M, ISG15, KLF2, and NLRP3 were among the other top-ranked genes. The sex-linked nature of the top FDR-significant genes suggests that a portion of the pseudobulk signal may be explained by demographic imbalance. Therefore, rather than being evidence of DFU-specific macrophage drivers, the DE data should be presented as candidate-prioritization evidence.

### 3.6 Volcano plot and enrichment analysis of DE genes

**Figure 7:**
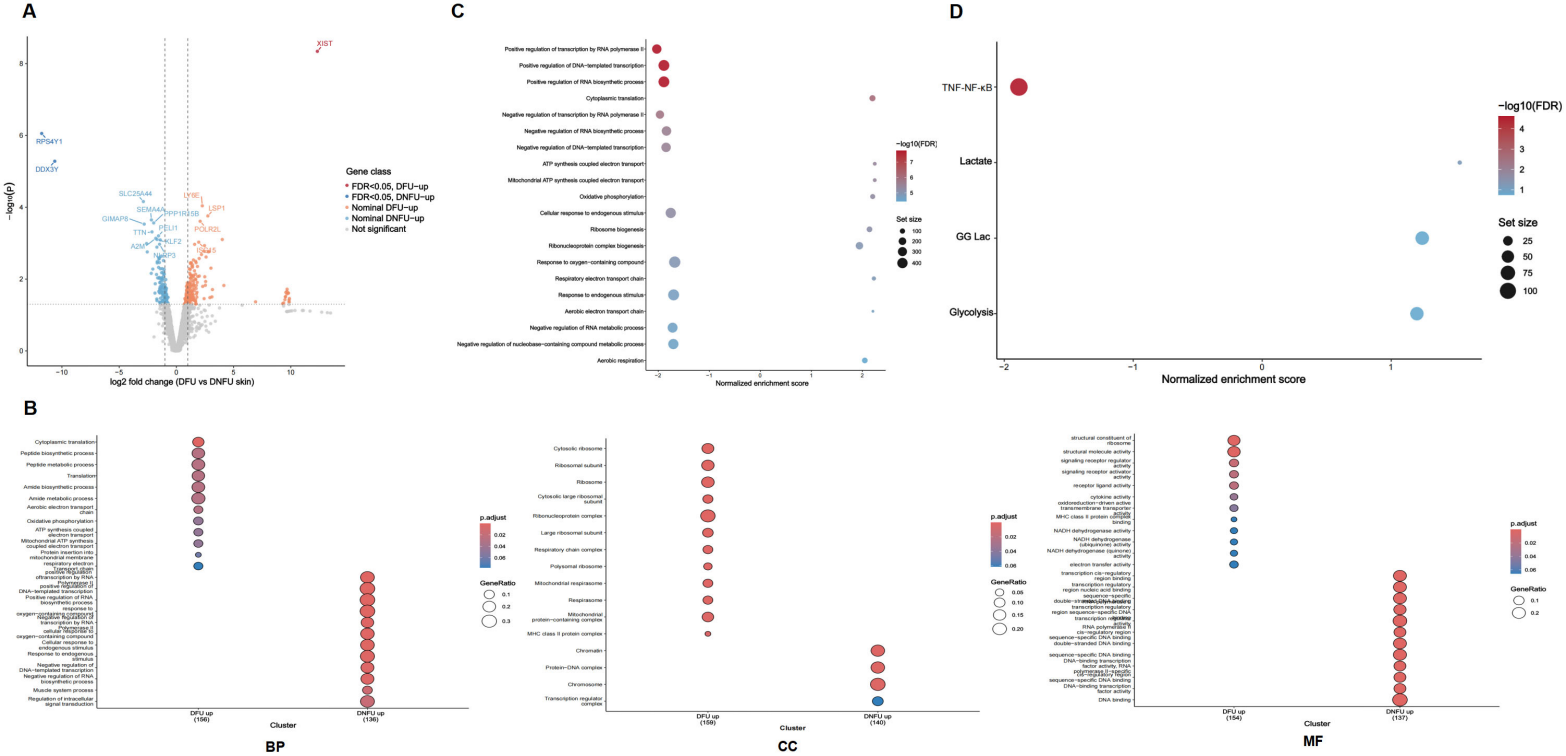
Volcano plot and enrichment analysis of macrophage pseudobulk differential-expression genes. Macrophage pseudobulk differential expression compared DFU with DNFU skin using edgeR quasi-likelihood testing in 7 eligible samples (2 DFU and 5 DNFU skin samples; minimum 15 macrophages per sample). **(A)** Volcano plot of macrophage differential-expression results. **(B)** GO Biological Process over-representation analysis of nominally changed macrophage genes. **(C)** GO Biological Process gene-set enrichment analysis of the full ranked macrophage differential-expression list. **(D)** Gene-set enrichment analysis of the custom UCell gene sets used in this study. Enrichment analyses are presented as exploratory functional summaries of the macrophage differential-expression ranking.

The distribution of 3,850 examined genes was summarized by the macrophage DE volcano graphic, which also emphasized the small number of FDR-significant genes (**Figure 7A**). 160 genes were categorized as DFU-up and 142 genes as DNFU-up using an exploratory nominal criterion of P < 0.05 and |logFC| >= 0.5. Nominal DFU-up genes were enriched for cytoplasmic translation (FDR = 4.06e-5), peptide biosynthesis (FDR = 0.0279), oxidative phosphorylation (FDR = 0.0465), and ATP production coupled electron transport (FDR = 0.0465), according to GO BP over-representation analysis (**Figure 7B**). The nominal DNFU-up genes were enriched for reactions to oxygen-containing substances (FDR = 1.04e-4), positive regulation of transcription by RNA polymerase II (FDR = 9.13e-6), and positive regulation of DNA-templated transcription (FDR = 9.13e-6).

GSEA of GO BP terms, performed on the full ranked list of differentially expressed genes in macrophages, revealed significant enrichment of pathways associated with DFU, including ATP synthesis coupled electron transport (normalized enrichment score [NES] = 2.24, FDR = 3.81e-6), cytoplasmic translation (NES = 2.20, FDR = 8.00e-7), oxidative phosphorylation (NES = 2.20), and antigen processing and presentation of peptide antigen (NES = 2.19, FDR = 2.00e-4) (**Figure 7C**). Conversely, pathways enriched in DNFU samples included positive regulation of transcription by RNA polymerase II (NES = -2.03, FDR = 1.80e-8), circadian rhythm (NES = -2.08, FDR = 4.88e-4), rhythmic process, and neuron projection guidance. Custom GSEA using UCell-derived gene sets demonstrated significant DNFU-directional enrichment of the TNF-NF-κB signaling pathway (NES = -1.89, FDR = 2.48e-5). In contrast, lactate metabolism showed only nominal enrichment that did not survive FDR correction (NES = 1.53, FDR = 0.062) (**Figure 7D**). Importantly, all these gene-level enrichment results should be interpreted as exploratory and do not supersede the sample-level UCell analysis, which found no statistically significant differences in macrophage pathway scores after FDR adjustment.

### 3.7 CellChat differential communication analysis

**Figure 8:**
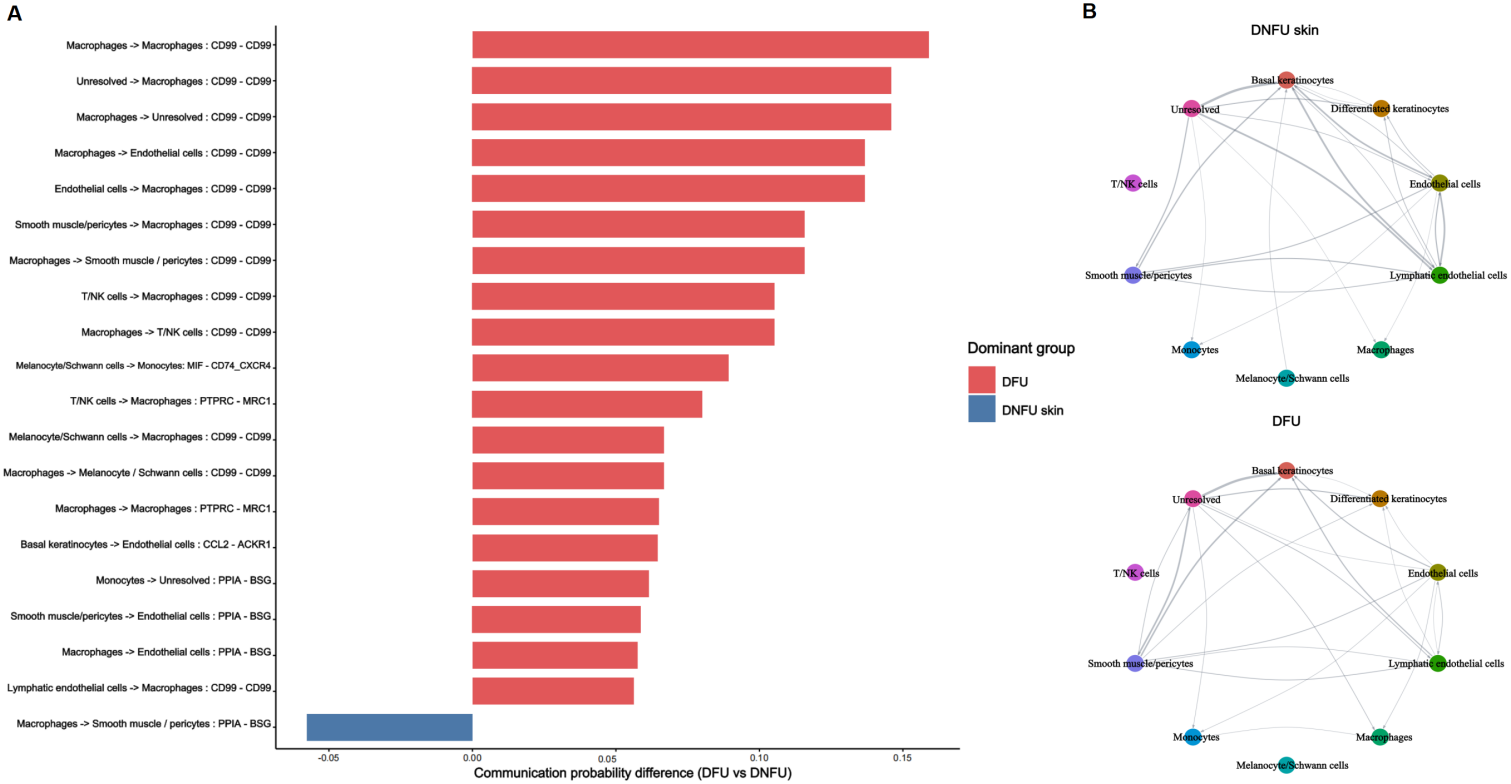
CellChat communication comparison. Cell-cell communication inference was performed with CellChat on the Harmony-integrated atlas comprising 5 DFU and 8 DNFU skin samples, with DFU and DNFU skin analyzed separately and then compared. Only cell populations shared by both groups and represented by at least 60 cells per condition were retained, resulting in 10 included cell populations. **(A)** Top differential ligand-receptor pairs ranked by communication probability difference between DFU and DNFU skin, highlighting macrophage-linked interactions. **(B)** CellChat network comparison between DNFU skin and DFU based on inferred communication probabilities among the retained cell populations.

Differential cell-cell communication analysis using CellChat identified macrophage-associated ligand-receptor pairs as the most prominent interactions enriched in DFU tissues (**Figure 8A**). CD99-CD99 interactions involving macrophages were among the strongest DFU-upregulated communication events, encompassing macrophage-macrophage, macrophage-endothelial, endothelial-macrophage, macrophage-smooth muscle/pericyte, and macrophage-T/NK cell crosstalk. The MIF-CD74/CXCR4 signaling axis was also identified as a top DFU-associated communication signal (**Figure 8B**). While these findings do not establish functional intercellular signaling *in vivo*, they nominate key macrophage-centered communication axes for subsequent spatial transcriptomic validation and functional experimental interrogation.

## 4 Discussion

This reanalysis comparing DFU and DNFU skin provides a rigorously controlled single-cell transcriptomic comparison between ulcerated diabetic foot tissue and non-ulcerated diabetic foot skin. Our findings comprise three macrophage-associated association-level signals that establish a foundational framework for future mechanistic investigations: (1) DFU-enriched macrophage subclusters representing potentially novel disease-associated cellular states; (2) reproducible pseudobulk transcriptomic signatures that prioritize candidate genes for prioritization; and (3) macrophage-mediated CD99-CD99 and MIF-CD74/CXCR4 axes that may mark or contribute to aberrant intercellular crosstalk in the wound microenvironment. While these findings do not yet establish macrophages as primary drivers of DFU pathogenesis nor detect FDR-significant global pathway activation, they generate actionable, testable hypotheses that warrant validation in larger, independent patient cohorts.

Cell composition analyses yielded modest results, limited to nominal trends that did not survive multiple-testing correction. While the observed nominal depletion of lymphatic endothelial cells and proportional shifts in immune and stromal cell populations may reflect tissue-context-specific alterations in DFU, the absence of statistically significant cell-type fraction differences after FDR correction precludes their interpretation as definitive diagnostic or prognostic biomarkers. Furthermore, no FDR-significant differences were detected in macrophage UCell pathway scores for glycolysis, lactate metabolism, TNF-NF-κB, or insulin/IGF signaling signatures. Notably, this apparent discrepancy with prior reports likely stems from fundamental differences in control group selection: most previous studies compared DFU tissue to healthy non-diabetic skin, which inherently exhibits distinct metabolic and immune profiles compared to diabetic skin (Aitcheson et al., 2021; Li et al., 2022; Jiang et al., 2024; Hu et al., 2025; Liu et al., 2025). In contrast, our study employed rigorously DNFU skin as the control group, which already harbors the baseline metabolic and inflammatory perturbations characteristic of diabetes mellitus (Theocharidis et al., 2020). This disease-relevant control design effectively subtracts the confounding effects of systemic diabetes, revealing that the aforementioned pathway alterations may represent generalized diabetic skin changes rather than ulcer-specific pathological events. Protein-level validation of the identified pathway signatures, including TNF-NF-κB signaling, represents a logical and essential next step for future mechanistic studies.

Nevertheless, macrophage subclustering analysis suggests that DFU tissue may harbor a distinct macrophage state composition compared to DNFU skin. This finding is consistent with extensive literature demonstrating that macrophages act as central orchestrators of inflammation, tissue repair, angiogenesis, matrix remodeling, and intercellular communication with wound-resident cells (Boniakowski et al., 2017; Wolf et al., 2021; Al Sadoun, 2022). However, consistency with established biological principles does not constitute causal proof. Therefore, the current subcluster findings should be used to guide marker selection and spatial localization experiments rather than to assert a definitive macrophage-driven pathogenic mechanism.

The pseudobulk transcriptomic results provide valuable prioritization of candidate genes but also reveal an important limitation of our study. The most significantly FDR-corrected differentially expressed genes, including XIST, RPS4Y1, and DDX3Y, are sex-linked, indicating that unbalanced demographic covariates may influence the macrophage transcriptional signature. For this reason, immune- and stress-associated candidates such as LY6E, LSP1, SEMA4A, PELI1, A2M, ISG15, KLF2, and NLRP3 should be considered exploratory and require validation in independent cohorts that are rigorously matched for sex, age, ulcer duration, infection status, vascular disease, and prior treatment exposure.

Volcano plot and gene set enrichment analyses provide a pathway-level summary of macrophage differential expression, but their interpretation requires careful consideration. DFU-directional enrichment of translation, oxidative phosphorylation, electron transport, and antigen presentation terms may reflect altered macrophage metabolic and immune processing states, whereas DNFU-directional enrichment of transcriptional regulation and rhythmic process terms may indicate alternative macrophage regulatory programs. However, because these analyses were performed on a small pseudobulk sample set and used nominal differential expression thresholds for over-representation analysis, they should be viewed as hypothesis-generating rather than confirmatory. The DNFU-directional TNF-NF-κB GSEA result also illustrates an important distinction: ranked GSEA tests for coordinated expression shifts across an entire gene list, whereas UCell analysis compares per-sample macrophage signature scores and did not detect FDR-significant group differences.

CellChat analysis prioritized macrophage-associated CD99-CD99 and MIF-CD74/CXCR4 interactions. These findings are valuable as they translate cell-type-resolved transcriptomic data into testable intercellular communication hypotheses. However, ligand-receptor inference from scRNA-seq data does not establish spatial proximity, protein abundance, receptor activation, or functional consequences *in vivo*. Spatial transcriptomics, multiplex immunostaining, proximity ligation assays, and targeted perturbation experiments are therefore required to determine whether these predicted interactions are functionally operative in DFU tissue.

This study has several important limitations. First, our analysis utilized a modest number of publicly available samples from a single dataset and lacked complete clinical covariate data (e.g., diabetes duration, wound chronicity, comorbidities, and demographic details), which limits the assessment of population heterogeneity and generalizability. Second, while DNFU skin represents a disease-relevant diabetic control, it does not substitute for healthy skin, acute wound tissue, or healing DFU tissue; thus, the observed DFU-associated signals cannot be conclusively designated as non-healing-specific versus general wound responses. Third, pathway scoring and CellChat analyses provide transcriptomic estimates rather than direct measurements of protein expression, post-translational modification, or functional activity. Consequently, all reported molecular associations remain correlative, and dedicated functional experiments (e.g., *in vitro* macrophage–stroma co-cultures, *in vivo* wound models, and genetic perturbation of the candidate ligand–receptor pairs) are required to establish causality. Fourth, pseudobulk analysis may be confounded by demographic imbalance between study groups, as evidenced by the detection of sex-linked genes among the few FDR-significant differentially expressed transcripts. Finally, we focused our analysis on macrophages because they yielded the most robust downstream signals in the current dataset; however, DFU healing is inherently a multicellular process involving keratinocytes, fibroblasts, endothelial cells, lymphatic cells, neurons, and multiple immune cell populations. Future studies integrating spatial transcriptomics, proteomics, and larger multicenter cohorts with well-annotated clinical metadata will be essential to validate and extend these findings.

## 5 Conclusion

The present single-cell reanalysis comparing DFU and DNFU skin supports a cautious, macrophage-associated interpretation rather than a definitive macrophage-driven pathogenic mechanism. UCell pathway scoring revealed no statistically significant differences in macrophage pathway activity between groups after FDR correction. The most robust and defensible findings comprise shifts in DFU-enriched macrophage subclusters, exploratory pseudobulk transcriptomic differences in macrophages, and macrophage-centered intercellular communication hypotheses identified via CellChat analysis. Collectively, these findings provide a prioritized list of candidate genes and pathways for future validation studies but do not establish a causal role for macrophages in DFU pathogenesis.

## Data Availability

The data presented in the study are available in the NCBI GEO repository, accession numbers GSE165816.

## Supplementary material

Supplementary Figure 1 Quality control of DFU and DNFU skin cells. **(A)** Violin plots of detected genes. **(B)** mitochondrial transcript percentages after QC.
