# Supplementary figures and images for: Single-cell reanalysis identifies macrophage-associated transcriptional and intercellular communication features in diabetic foot ulcers
Source: Front Cell Infect Microbiol. 2026 Jul 1;16:1875481. doi: 10.3389/fcimb.2026.1875481 (PMC13368467; doi:10.3389/fcimb.2026.1875481)

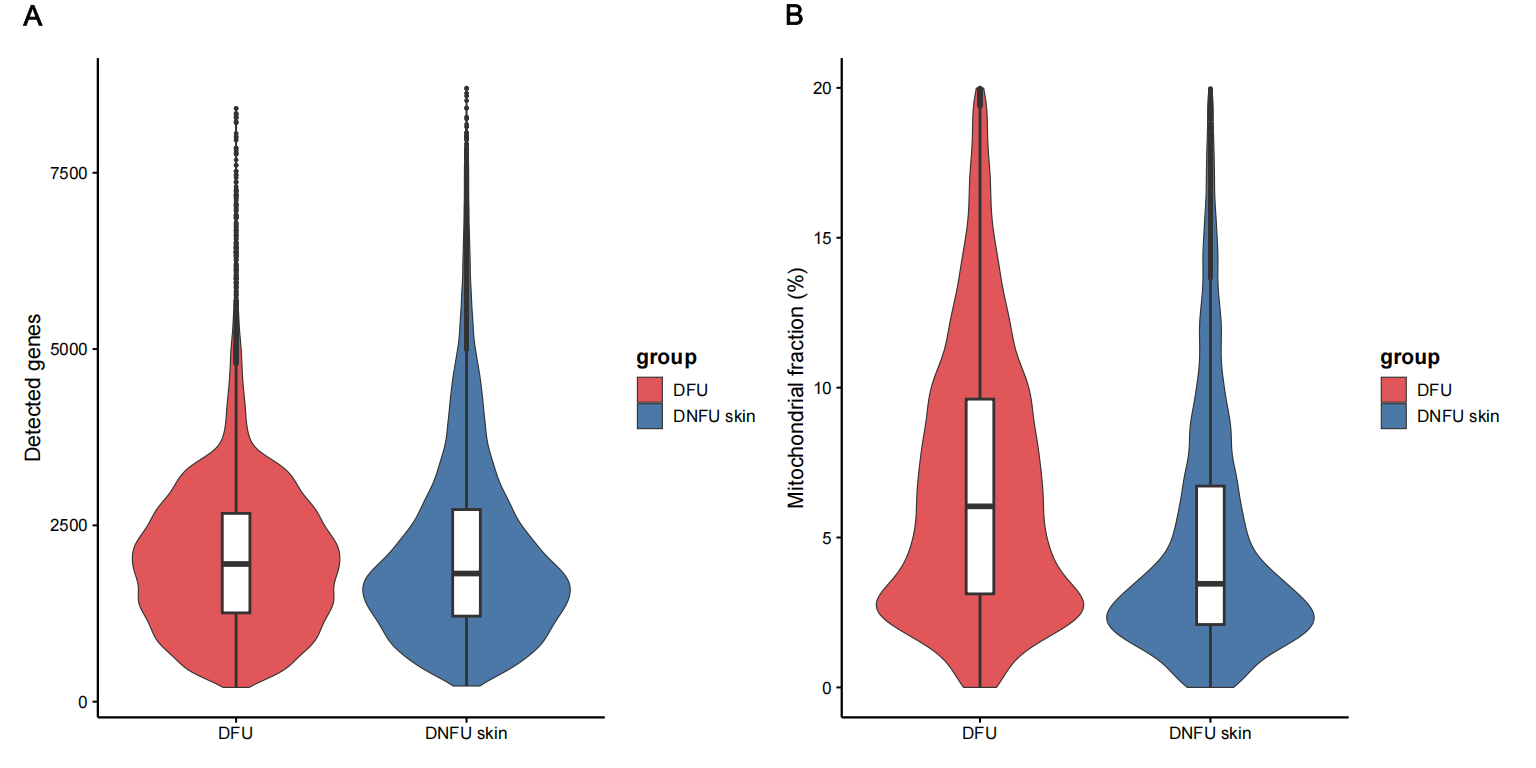

Supplement: Supplementary Figure 1 — Quality control of DFU and DNFU skin cells. (A) Violin plots of detected genes. (B) mitochondrial transcript percentages after QC. [file Image1.tif]
